# A vacuum ultraviolet laser with a submicrometer spot for spatially resolved photoemission spectroscopy

**DOI:** 10.1038/s41377-021-00463-3

**Published:** 2021-01-21

**Authors:** Yuanhao Mao, Dong Zhao, Shen Yan, Hongjia Zhang, Juan Li, Kai Han, Xiaojun Xu, Chuan Guo, Lexian Yang, Chaofan Zhang, Kun Huang, Yulin Chen

**Affiliations:** 1 College of Advanced Interdisciplinary Studies, National University of Defense Technology, Changsha, Hunan 410073 China; 2 Department of Optics and Optical Engineering, University of Science and Technology of China, Hefei, Anhui 230026 China; 3 College of Intelligence Science and Technology, National University of Defense Technology, Changsha, Hunan 410073 China; 4 State Key Laboratory of Low Dimensional Quantum Physics, Department of Physics, Tsinghua University, Beijing, 100084 China; 5 Department of Physics, Clarendon Laboratory, University of Oxford, Oxford, UK; 6 School of Physical Science and Technology, ShanghaiTech University, Shanghai, 201210 China

**Keywords:** Fluorescence spectroscopy, Micro-optics

## Abstract

Vacuum ultraviolet (VUV) lasers have demonstrated great potential as the light source for various spectroscopies, which, if they can be focused into a small beam spot, will not only allow investigation of mesoscopic materials and structures but also find application in the manufacture of nano-objects with excellent precision. In this work, we report the construction of a 177 nm VUV laser that can achieve a record-small (~0.76 μm) focal spot at a long focal length (~45 mm) by using a flat lens without spherical aberration. The size of the beam spot of this VUV laser was tested using a metal grating and exfoliated graphene flakes, and we demonstrated its application in a fluorescence spectroscopy study on pure and Tm^3+^-doped NaYF_4_ microcrystals, revealing a new emission band that cannot be observed in the traditional up-conversion process. In addition, this laser system would be an ideal light source for spatially and angle-resolved photoemission spectroscopy.

## Introduction

The rapid development of two-dimensional quantum materials, such as twisted bilayer graphene^1,2^, monolayer room-temperature ferromagnetic materials^3^, monolayer copper superconductors^4^, quantum spin Hall materials^5^, and transition metal dichalcogenide heterostructures^6,7^, has demonstrated both important scientific implications and promising application potential. To characterize the electronic structure of these materials/devices, angle-resolved photoemission spectroscopy (ARPES) is commonly used to measure the energy and momentum of electrons photoemitted from samples illuminated by X-ray or vacuum ultraviolet (VUV) light sources. Although the X-ray-based spatially resolved ARPES has the highest spatial resolution (~100 nm)^8–11^ benefitting from the relatively high photon energy (therefore short wavelength), its energy resolution is typically mediocre (>10 meV), which makes it difficult to visualize the fine details of the electronic structure in many novel quantum materials, such as those mentioned above. Complementary to X-ray light sources, VUV laser-based light sources can offer much better energy resolution (~0.2 meV^12^), deeper depth of detection and lower cost (compared to synchrotron light sources) and therefore have recently drawn increasing attention^13–16^. However, the longer wavelength of the VUV light source also deteriorates its spatial resolution (typically several micrometers to date), making it insufficient for characterizing small-size flake samples or spatially inhomogeneous (e.g., magnetic, electronic or composite domain) materials.

Persistent efforts have been made to reduce the focal spot size since the first development of VUV-based ARPES in 2008^17^. Laser-based scanning ARPES with a spatial resolution of 5–10 μm was reported for a tuneable wavelength range of 191–210 nm^16^. Recently, a spatial resolution of VUV-based ARPES down to 3 μm was achieved by using a higher numerical-aperture (NA) lens at the 206 nm wavelength (6.0 eV)^15^. Although the VUV laser with higher photon energy (e.g., 7 eV carried by a 177 nm-wavelength photon) could help the ARPES measurement cover a larger momentum space, the current 7 eV VUV-based ARPES has a much poorer spatial resolution (~several tens of micrometers^18^) for the following reasons:

First, severe spherical aberration exists in a high-NA refraction lens. Second, only very limited materials can be used in optics for correcting the spherical aberration due to the strong absorption at VUV frequencies. Third, it is practically difficult to check the quality (collimation, uniformity and efficient diameter) of the incident beam and the alignment among optical elements, as the VUV beam is invisible and all optics have to be placed in vacuum or a sealed chamber filled with inert gas.

In this work, by overcoming various challenges, we report a 177 nm VUV laser light source for scanning photoemission microscopy with a focal spot of ~0.76 μm by using a spherical-aberration-free zone plate. The size of the focal spot was measured by a standard knife-edge scan, and the sub-μm size was further demonstrated by running realistic scans on micrometer-period metal stripe arrays and an exfoliated graphene flake. Based on this microscopy, we also built an off-axis fluorescence detection platform that exhibits superior capability to conventional laser systems in revealing subtle features of materials.

## Results

### Design and fabrication of a VUV flat lens

**Fig. 1:**
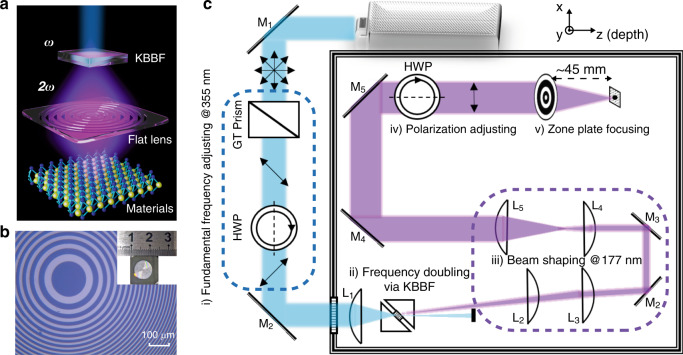
Working principle of the proposed VUV-based photoemission microscopy. **a** Illustration of the laser beam going through the KBBF crystal (top) and the flat lens (middle); **b** microscopic image of the flat lens etched on a CaF_2_ substrate (insert: photo of the optical device); **c** schematic optical setup of the laser focusing system including five components: (i) fundamental frequency adjustment (of the polarization and power), (ii) frequency doubling, (iii) beam shaping, (iv) polarization adjustment, and (v) focusing of light. GT prism Glan–Taylor prism, HWP half-wave plate, L lens, M mirror

To avoid the spherical aberration, we introduce planar diffractive lenses^19–22^ that can realize tight focusing of light by fine tuning of the interference from multiple beams. In these lenses, the carefully designed micro/nanostructures are responsible for tailoring the amplitude or phase of each beam so that the constructive interference achieved at the target plane guarantees a well-confined hotspot without the spherical aberration being involved. Considering the lack of high-refractive-index and lossless materials^23,24^ at VUV wavelengths, we utilize a binary-amplitude zone plate (see Fig. 1a) that has radii of *r*_*n*_ = [*nλf* + (*nλ*/2)^2^]^1/2^, where *n* (= 0, 1, 2, …, *N*) denotes the index of the transparent belt circles, the operating wavelength *λ* equals 177 nm, and the focal length *f* of the lens is taken as 45 mm in this work. The dimensions of the zone plate are derived mathematically to realize the purpose of constructive interference, thereby avoiding the spherical aberration in this flat lens. Due to the limited size of the chamber of our laser system, the diameter of the flat lens is set to be ~1 cm, leaving *N* = 3000 and *r*_*N*_ = 4.895 mm. The flat lens is fabricated onto a chrome film sitting on a CaF_2_ substrate by using a laser writing tool (AdvanTools, ATD1500), followed by a dry etching process. A microscopic image of the fabricated lens is shown in Fig. 1b, where the uniformly distributed rings indicate good fabrication.

### 177 nm/7 eV VUV laser system

Figure 1c sketches our VUV laser focusing system containing five functional parts: a 355 nm laser, a second-harmonic generation stage, a beam shaping stage, a polarization adjustment part, and a focusing element of the flat lens. A commercial pulsed laser with a center wavelength of 355 nm and a repetition rate of 120 MHz is used as the pumping source for second harmonic generation (SHG). To maximize the SHG conversion efficiency, a Glan–Taylor prism and a half-wave plate are used to adjust the polarization and the power of the 355 nm beam that is focused into a potassium fluoroboratoberyllate (KBBF) nonlinear crystal by a convex lens. When the phase-matching condition is met, the conversion efficiency can reach 2.05%^25^.

Due to the strong absorption of 177 nm photons in air, all optics, including the KBBF crystals, are placed in a chamber either evacuated or filled with high-purity inert gas. To enhance the transmission efficiency, all lenses are made of CaF_2_ that is transparent at the 177 nm wavelength. The SHG light emits at a walk-off angle of approximately 9 degrees and is well separated from the residual 355 nm laser that is stopped by an optical dump. To modify the elliptical beam profile caused by anisotropic divergence of SHG light through the KBBF crystal, a set of cylindrical and spherical lenses are used as the beam shaping system, leaving a nearly collimated and quasi-circular beam. Then, a rotatable half-wave plate is applied to tune the polarization of light (which will facilitate its future use as an ARPES light source). Finally, the FZP is employed to directly focus the 177 nm laser beam, with a measured transmission of more than 20%, which is significantly enhanced compared with extreme-UV zone plates^26,27^. By measuring the focusing efficiency of ~40% (Section 5 in Supplementary Materials), we can calculate the optical efficiency of this flat FZP lens as 8%, which approaches the theoretical efficiency limit of 10% for a binary-amplitude optical element. For the beam-profile characterization described below, the knife edge, metal array, and graphene flakes are all located at the focal plane, followed by an optoelectronic detector for beam power measurements.

### Beam spot profile

**Fig. 2:**
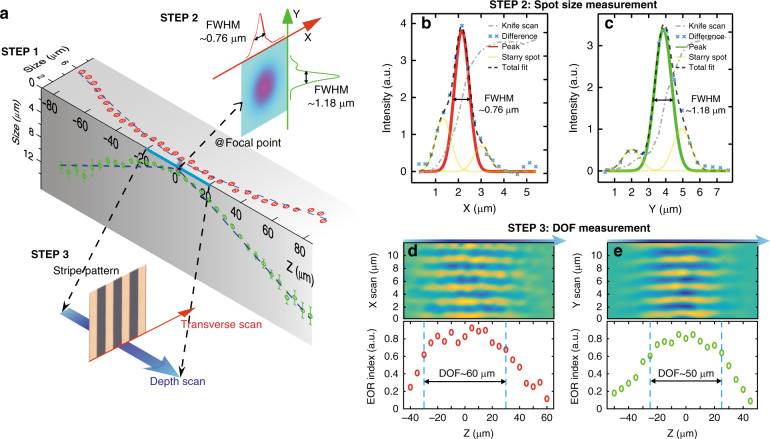
Characterizing the optical performance of our flat lens. **a** Measurement of the focal spot. The experimental profiles of focal spots near the focal plane are measured by knife-edge scanning. Based on the profiles at the different *z*-cut planes, the lateral (*x*- and *y*-direction) intensity profiles of the real spot are retrieved by our homemade algorithm and then yield the spot size (FWHM) labeled by red (*x*-direction) and green (*y*-direction) circles. **b**, **c** Calculation of the spot sizes in the *x*-direction **b** and *y*-direction **c** based on the multi-Gaussian fitting method. **d**, **e** Depth of focus (DOF) in the *x*-direction **d** and *y*-direction **e**. Transversal (i.e., in the grating direction) scanning of a grating pattern is employed to measure the DOF along the depth, as sketched by Step 3 in **a**. The upper images display the evolution of the intensity distribution obtained in the laser transmission scan of the grating pattern. A small spot yields clear stripes along the *x*- or *y*-direction, while a large spot gives a uniform intensity. The lower images plot the effective optical resolution (EOR) index of the laser spot along the depth so that we can evaluate the DOF of the lens

Due to imperfect beam shaping, the 177 nm laser incident on the FZP is not perfectly circular, yielding an elliptical spot at the focal plane. The lateral (*x*- and *y*-direction) dimensions of the focal spot are measured via a knife-edge scanning method with a step of 10 nm. The focal length is determined by rough (10 μm step) and fine (~100 nm step) scanning along the propagation direction of light. Both scans give nearly identical focal lengths of approximately 45 mm, which offers a long working distance between the focal point and the FZP, enabling off-axis detection without steric hindrance during the photoemission process and spectroscopic measurements. Figure 2a shows the profile of the focal spot from experiments. Due to the distortion from the CaF_2_ optical elements^28,29^, the focal spot is accompanied by satellite bands. In Fig. 2b, c, the derivative of the scanned beam power with respect to *x* or *y* is used to show the experimental focus profile of the focal spot. Then, a multi-Gaussian fitting method is employed to eliminate the influence of the satellite bands, resulting in a spot size of ~0.76 and ~1.18 μm along the *x*- and *y*-directions, respectively. The size of the experimental focal spot approaches the theoretical value of 0.8 μm, which confirms the validity of our FZP design. Since the power of the 177 nm laser is above 1 mW (~10^15^ photons/s) and the focal spot has an area of 2.82 (≈0.76 × 1.18π) μm^2^, we can evaluate the brightness by using the power density of 355 MWm^−2^, which is sufficient for ARPES^12^. In addition, the experimental results show that the focused beam has a moderately larger divergence along the *y* direction than along the *x* direction (Fig. 2a), which originates mainly from the inherent divergence of the incident beam.

The depth of focus (DOF) is one of the key parameters to evaluate the tolerance of the system to out-of-focus conditions. From the viewpoint of practical applications, the DOF in this work is derived based on the ability to resolve a metal stripe array with a half-pitch of 1 μm, i.e., whether the metal stripe array can be spatially resolved by scanning the focal spot, which is different from the commonly defined DOF of a focusing lens. To measure the DOF, the focal spot is used to transversally scan a standard CaF_2_-based binary grating formed by the metal stripe array (see Step 3 in Fig. 2a) placed at different positions along the propagation direction of light. The light transmitted through the grating is recorded by the detector. The experimental results for measuring the DOF are shown in Fig. 2d, e, where the pseudo-color images in the upper panel represent the transmitted intensity collected by the detector over the cross-section planes of *X*–*Z* and *Y*–*Z*. When the sample is located within the DOF, the transmitted power shows a clear oscillation with the period of the grating. In contrast, in the off-focus regime, the oscillation smears and eventually disappears with the increasing size of the off-focus illuminating beam spot. Theoretically, when the illuminating spot is larger than 2 μm, gratings with a 1 μm half-pitch cannot be substantially distinguished.

To further examine the DOF quantitatively, we introduce an evaluation method of the effective optical resolution index (EOR index), which is calculated as follows:

1 $$EO{R_{Index}}({z_0}) = \,\frac{{{\text{FT}}\{ {scan(x,{z_0})}\} \left|_{\omega = \frac{1}{T}} \right. - \min \{ {scan(x,{z_0})}\}}}{{\max \{ {scan(x,{z_0})}\} - \min \{ {scan(x,{z_0})}\} }}$$

where *z*_0_ is the distance between the surface of the stripe array and the focal point, the function scan (*x, z*_0_) is the intensity distribution curve obtained by a transverse scan of the stripe pattern at *z*_0_, *T* is the period of the stripe pattern (2 μm in our case), and FT{ } stands for the Fourier transform. Based on the distinguishable stripes in the recorded intensity of Fig. 2d, e, we use EOR = 0.6 as the threshold value, above which the sample is still located within the DOF. The calculated EOR indices are shown in the bottom panels of Fig. 2d, e, which indicate that the calculated DOFs along the *x*- and *y*-directions are 60 and 50 μm, respectively. The long DOF is sufficient to characterize quantum materials with a high spatial resolution for most spectroscopies.

### High-resolution imaging

**Fig. 3:**
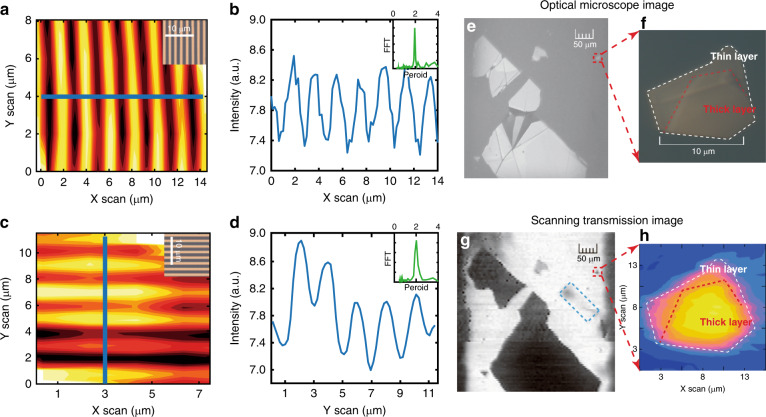
Scanning images of microscale gratings and graphene. **a**, **b** Two-dimensional (**a**) and line (**b**) scan images of a 2 μm-period grating with a vertical orientation. The intensity profile shown in **b** is extracted along the solid line in **a**. The insert in **a** shows a microscopic image of the grating, while the insert in **b** gives the fast Fourier transform of the line-scan intensity. **c**, **d** Two-dimensional (**c**) and line (**d**) scan images of a 2 μm-period grating with a horizontal orientation. The intensity profile shown in **d** is extracted along the solid line in **c**. The insert in **c** shows a microscopic image of the grating, while the insert in **d** gives the fast Fourier transform of the line-scan intensity. **e**–**h** Microscopic images (**e**, **f**) and scanning transmission images (**g**, **h**) of a graphene sample on a CaF_2_ substrate. The images in **f**, **h** are magnified images of the small graphene sample marked in **e**, **g**, respectively

To characterize the spatial resolution of the focal beam spot, we first carry out scans on both horizontally and vertically oriented gratings. The results are shown in Fig. 3a, c, where the microscopic images are provided in the insets as references. From both scans in Fig. 3a, c, a line cut is taken and shown in Fig. 3b, d, respectively, clearly showing the expected period of 2 μm, as also confirmed by the Fourier transformation (see the insets in Fig. 3b, d). These results indicate that the feature of the 1 μm half-pitch can be well resolved by our setup. Considering the high contrast in the scanned images, the practical spatial resolution is better than 1 μm, showing an enhancement of three times compared with previous results^15^.

To test the imaging ability of our setup for real materials, we use an exfoliated graphene sample on a CaF_2_ substrate (see its microscopic image in Fig. 3e). The scanning image of the graphene flakes obtained by our VUV scanning microscope (Fig. 3g) shows nice agreement with the optical microscope image (Fig. 3e) except for a region (enclosed by the blue-dashed rectangle in Fig. 3g) that arises from the residual adhesive used during the exfoliation process that is invisible in the optical microscope image.

Furthermore, an even smaller flake of graphene (marked by the red-dashed rectangle in Fig. 3e) is examined in more detail. In Fig. 3f, the microscope image of this flake shows a size of approximately 10 μm. The VUV laser scanning image in Fig. 3h not only captures the shape of the graphene flake but also vividly reveals the fine change in the thickness in terms of the gradient transmission. As the multilayer graphene sample has a similar size to other commonly exfoliated 2D monolayer flakes^4,14,15^, our focused VUV laser system can be readily used to investigate 2D exfoliated materials for a wide range of research works.

### Off-axis spectroscopy

**Fig. 4:**
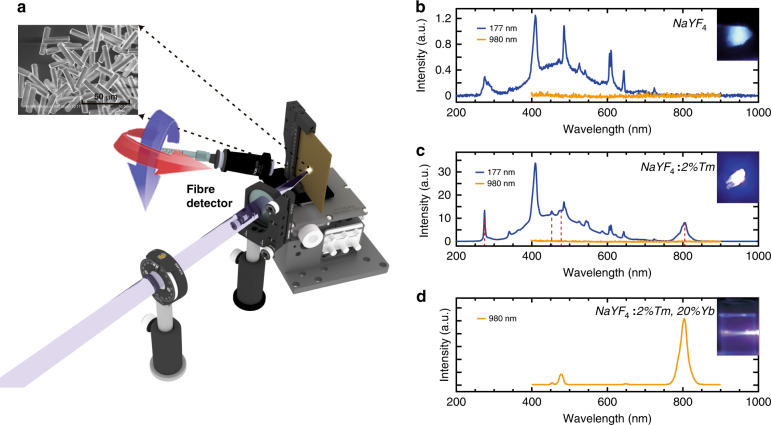
Off-axis spectroscopy. **a** Schematic illustration of off-axis VUV fluorescence spectroscopy with both reflection and transmission, where the inset is a scanning electron microscope (SEM) image of a typical sample with microcrystalline particles. **b**, **c** NaYF_4_ and NaYF_4_:2%Tm emission bands, respectively, under 177 and 980 nm excitation. **d** NaYF_4_:2%Tm,20%Yb emission bands under 980 nm excitation (Ref. ^31^)

In addition to the small-size beam spot, another advantage of the FZP is its ultralong focal length that enables off-axis spectroscopy without steric hindrance. As shown in Fig. 4a, the focused 177 nm laser beam is used to illuminate the sample in an off-axis system, and the reflected fluorescence signal can be collected by a fiber and recorded by a spectrometer. To validate this hypothesis, we examine the luminescence properties of pure (Fig. 4b) and Tm^3+^(trivalent thulium)-doped (Fig. 4c) hexagonal NaYF_4_ microcrystals, widely studied materials with high fluorescence emission efficiency.

Excited by the tightly focused 177 nm laser spot, NaYF_4_ and NaYF_4_:Tm (2 mol%) yield different emission bands, as shown in Fig. 4b–d. Typically, the bands at 277 nm (^3^P_1_ → ^3^H_6_), 450 nm (^1^D_2_ → ^3^F_4_), 475 nm (^1^G_4_ → ^3^H_6_), and 800 nm (^3^H_4_ → ^3^H_6_)^30–32^ correspond to the fluorescence from Tm^3+^. The remaining observed emission bands do not directly correspond to the atomic and ionic energy levels of Na, Y, or F and are attributed to the NaYF_4_ bandgap luminescence^32^. This result is out of expectations since the NaYF_4_ microcrystal is transparent under the excitation of frequently used near-infrared laser sources, such as 980 nm lasers^31,33^. Notably, we find that in the emission bands of NaYF_4_, the band around 280 nm^34^ and the broad band between 350 and 600 nm are partly derived from the CaF_2_ optical elements (see Supplementary materials for details). Moreover, compared to the up-conversion emission of Tm^3+^ ions (Fig. 4d)^31^, our reported emission of Tm^3+^ ions shows a new ultraviolet band at 277 nm (^3^P_1_ → ^3^H_6_)^30^.

This finding is unexpected because no sensitizer was doped into the host lattice, which is usually necessary to excite the activator when traditional fluorescence spectroscopy is used. For example, without sensitizers such as Yb^3+^ ions, none of these samples could produce fluorescence emission under the excitation source of a 980 nm continuous-wave laser (see Fig. 4b, c). Therefore, the 177 nm laser can directly detect the emission of pure NaYF_4_ and NaYF_4_:Tm (2 mol%) without the need for sensitizer doping, which significantly facilitates the preparation of samples and extends its capacity to characterize unexplored materials.

The off-axis setup allows the relative angle between the fiber detector and the sample to be easily tuned according to the user’s requirements, making it possible to study the angular distribution of the reflected signal from isotropic materials. When pumped by light with different polarizations, the system could simultaneously collect data with various degrees of freedom, such as angle, space and polarization. Therefore, it could work as a versatile system that could provide the full-scale data of various materials. Due to its high photon concentration and long focal length, the proposed system has another potential application of UV lithography to fabricate nanostructures in nanophotonics and quantum computing chips. In fact, the focal spot could be further reduced by increasing the numerical aperture of the flat lens, which, however, requires the challenging fabrication of a lens with large-scale dimensions and much smaller features simultaneously.

## Discussion

In summary, a VUV (177 nm) laser scanning system with a submicron spot of 0.76 μm, a relatively long working distance of 45 mm, and a DOF of ~50 μm has been proposed to characterize various materials, including gold and graphene. Benefiting from the long focal length, the system could also work as a VUV fluorescence spectroscopy system with both off-axis reflection and transmission. With the measured fluorescence of a Tm^3+^-doped hexagonal-phase NaYF_4_ microcrystal sample, we observe new emission bands compared with the conventional up-conversion emission, indicating the unique advantage in characterizing the material. The proposed VUV laser system can be re-equipped for usage in low-cost ARPES and might benefit quantum materials, condensed matter physics and nanophotonics.

## Materials and methods

### Fabrication of the flat lens

The designed lenses were fabricated using the standard fabrication technique of laser direct writing. The process began with the deposition of a 150-nm thick chromium film on a CaF_2_ substrate (15 mm × 15 mm × 2 mm) by sputtering (Kurt J. Lesker. LAB 18). Then, a 1.4-μm thick S1813 positive resist layer was spin-coated onto the chromium film at 4000 rpm and baked at 115 °C for 60 s. Next, the dried resist was directly patterned by ATD1500 using maskless lithography at 405 nm. After that, the device was developed in 2.5% TMAH (tetramethylammonium hydroxide) for 50 s at room temperature followed by the use of flowing DI water to reduce residue. After drying in a N_2_ atmosphere, the exposed chromium film without the resist hard mask was removed by an inductively coupled plasma-reactive ion etching (ICP-RIE) system (Oxford, Plasma Pro System100 ICP180). The ICP RF power was kept at 600 W, and the bias power was set at 10 W. The processing pressure was 10 mTorr with a Cl_2_ gas flow of 45 sccm and an O_2_ gas flow of 4 sccm. The helium pressure was 10 Torr for backside cooling, while the substrate temperature was maintained at 50 °C. Due to the good selectivity of the gas, we can easily etch the chromium film without destroying the CaF_2_ substrate. Finally, the residual positive resist was removed by NMP (N-methylpyrrolidone) solvent, which was heated in water at 80 °C for 15 min.

### Preparation of graphene flakes on a CaF_2_ substrate

A dry transfer procedure was applied during the preparation of monolayer graphene on a CaF_2_ substrate. The transfer handle was prepared by spin-coating polyvinyl alcohol (PVA) solution (4:96 ratio in DI water) on a layer of PDMS held by a glass slide. Using a six-dimensional manipulator, the handle was vertically aligned with graphene flakes exfoliated on a Si/SiO_2_ substrate in the field of a microscope. The PVA layer then detached from the handle after being brought into contact with the substrate and heated to 50 °C. The graphene/PVA layer could then be picked up by a tweezer and reattached to PDMS. Subsequently, the Si/SiO_2_ wafer was replaced by a pre-marked CaF_2_ substrate, and the graphene flakes were attached to the CaF_2_ substrate in exact alignment with the marked zone under microscopy. Repeating the heating process, graphene flakes along with PVA film were left on the CaF_2_ substrate. All the PVA residue could be theoretically eliminated by a DI water washing process following nitrogen gas rinsing.

### Preparation of microcrystals

Y(NO_3_)_3_·6H_2_O (99.9% metal basis), Tm(NO_3_)_3_·6H_2_O (99.9% metal basis), ethylenediamine tetraacetic acid disodium salt dihydrate (EDTA–2Na, analytical reagent), sodium hydroxide (NaOH, analytical reagent), and ammonium fluoride (NH_4_F, analytical reagent) were purchased from Aladdin Industrial Corporation. All the chemicals were used as received without further purification.

β-NaYF_4_:xTm (*x* = 0, 2 mol%) microcrystals were synthesized by a facile hydrothermal method. In a typical procedure, EDTA-2Na (1 mmol) and NaOH (6 mmol) were mixed with 13.5 mL deionized water under constant stirring in a flask to form a clear solution. Then, 5 mL of an aqueous solution including a stoichiometric amount of Ln(NO_3_)_3_ (0.2 M) was added into the solution under vigorous stirring. Subsequently, 8 mL of NH_4_F (2.0 M) aqueous solution and 7 mL of dilute hydrochloric acid (1 M) were injected into the flask. After stirring for 1.5 h, the mixture was transferred to a 50 mL Teflon-lined autoclave, heated at 220 °C for 40 h, and then slowly cooled to room temperature. After the reaction, the system was cooled to room temperature naturally, and the precipitates were collected and centrifuged several times with distilled water and ethanol and finally dried at 60 °C for 10 h in air.

## Supplementary information

Supplementary Information

## Supplementary information

**Supplementary information** The online version contains supplementary material available at 10.1038/s41377-021-00463-3.

## References

[1] Cao Y (2018). Correlated insulator behaviour at half-filling in magic-angle graphene superlattices. Nature.

[2] Cao Y (2018). Unconventional superconductivity in magic-angle graphene superlattices. Nature.

[3] Deng YJ (2018). Gate-tunable room-temperature ferromagnetism in two-dimensional Fe_3_GeTe_2_. Nature.

[4] Yu YJ (2019). High-temperature superconductivity in monolayer Bi_2_Sr_2_CaCu_2_O_8+δ_. Nature.

[5] Tang SJ (2017). Quantum spin Hall state in monolayer 1T’-WTe_2_. Nat. Phys..

[6] Van Der Zande AM (2014). Tailoring the electronic structure in bilayer molybdenum disulfide via interlayer twist. Nano Lett..

[7] Zhang Z (2020). Flat bands in twisted bilayer transition metal dichalcogenides. Nat. Phys..

[8] Dudin P (2010). Angle-resolved photoemission spectroscopy and imaging with a submicrometre probe at the SPECTROMICROSCOPY-3.2L beamline of elettra. J. Synchrotron Radiat..

[9] Hoesch M (2017). A facility for the analysis of the electronic structures of solids and their surfaces by synchrotron radiation photoelectron spectroscopy. Rev. Sci. Instrum..

[10] Avila J, Asensio MC (2014). First NanoARPES user facility available at SOLEIL: an innovative and powerful tool for studying advanced materials. Synchrotron Radiat. N..

[11] Rotenberg E, Bostwick A (2014). microARPES and nanoARPES at diffraction-limited light sources: opportunities and performance gains. J. Synchrotron Radiat..

[12] Zhou XJ (2018). New developments in laser-based photoemission spectroscopy and its scientific applications: a key issues review. Rep. Prog. Phys..

[13] Nguyen PV (2019). Visualizing electrostatic gating effects in two-dimensional heterostructures. Nature.

[14] Mo SK (2017). Angle-resolved photoemission spectroscopy for the study of two-dimensional materials. Nano Convergence.

[15] Cucchi I (2019). Microfocus laser–angle-resolved photoemission on encapsulated mono-, Bi-, and few-layer 1T′-WTe_2_. Nano Lett..

[16] Iwasawa H (2017). Development of laser-based scanning µ-ARPES system with ultimate energy and momentum resolutions. Ultramicroscopy.

[17] Liu GD (2008). Development of a vacuum ultraviolet laser-based angle-resolved photoemission system with a superhigh energy resolution better than 1 meV. Rev. Sci. Instrum..

[18] Ping, Ai. et al. Development of angle-resolved photoemission spectroscopy technique and its study on high-temperature cuprate superconductors [Thesis]. University of Chinese Academy of Sciences (2019).

[19] Huang K (2014). Optimization-free superoscillatory lens using phase and amplitude masks. Laser Photonics Rev..

[20] Huang K (2018). Planar diffractive lenses: fundamentals, functionalities, and applications. Adv. Mater..

[21] Huang K (2015). Ultrahigh-capacity non-periodic photon sieves operating in visible light. Nat. Commun..

[22] Qin F (2017). A supercritical lens optical label-free microscopy: sub-diffraction resolution and ultra-long working distance. Adv. Mater..

[23] Huang K (2019). Ultraviolet metasurfaces of ≈80% efficiency with antiferromagnetic resonances for optical vectorial anti-counterfeiting. Laser Photonics Rev..

[24] Li J (2019). Resonance-free ultraviolet metaoptics via photon nanosieves. Opt. Lett..

[25] Xu B (2015). Generation of high power 200 mW laser radiation at 177.3 nm in KBe_2_BO_3_F_2_ crystal. Appl. Phys. B.

[26] Rösner B (2019). Zone plates for angle-resolved photoelectron spectroscopy providing sub-micrometre resolution in the extreme ultraviolet regime. J. Synchrotron Radiat..

[27] Koch RJ (2018). Nano focusing of soft X-rays by a new capillary mirror optic. Synchrotron Radiat. N..

[28] Marsh ER (2005). Predicting surface figure in diamond turned calcium fluoride using in-process force measurement. J. Vac. Sci. Technol. B Microelectron. Nanometer Struct. Process Meas. Phenom..

[29] Zong HW (2019). Development of research on damage characteristics of calcium fluoride crystal under deep ultraviolet laser irradiation. Laser Optoelectron. Prog..

[30] Dong H, Sun LD, Yan CH (2015). Energy transfer in lanthanide upconversion studies for extended optical applications. Chem. Soc. Rev..

[31] Yi GS, Chow GM (2006). Synthesis of hexagonal-phase NaYF_4_: Yb, Er and NaYF_4_: Yb, Tm nanocrystals with efficient up-conversion fluorescence. Adv. Funct. Mater..

[32] Huang BL (2016). Fundamental view of electronic structures of β-NaYF_4_, β-NaGdF_4_, and β-NaLuF_4_. J. Phys. Chem. C..

[33] Nyk M (2008). High contrast in vitro and in vivo photoluminescence bioimaging using near infrared to near infrared up-conversion in Tm^3+^ and Yb^3+^ doped fluoride nanophosphors. Nano Lett..

[34] Mikhailik VB (2006). Scintillation properties of pure CaF_2_. Nucl. Instrum. Methods Phys. Res. Sect. A: Accelerators Spectrometers Detect. Associated Equip..

