# Supplementary material for: A vacuum ultraviolet laser with a submicrometer spot for spatially resolved photoemission spectroscopy
Source: Light Sci Appl. 2021 Jan 21;10:22. doi: 10.1038/s41377-021-00463-3 (PMC7820001; doi:10.1038/s41377-021-00463-3)
Supplement: Supplementary file 1 — Supplementary Information [file 41377_2021_463_MOESM1_ESM.docx]

**Supplementary Information for**

A Vacuum-ultraviolet Laser with Sub-micrometer Spot for Spatially Resolved Photoemission Spectroscopy

Yuanhao Mao^1,#^, Dong Zhao^2,#^, Shen Yan^1^, Hongjia Zhang^3^, Juan Li^2^, Kai Han^1^, Xiaojun Xu^1^, Chuan Guo^1^, Lexian Yang^4^, Chaofan Zhang^1,*^, Kun Huang^2,*^, Yulin Chen^4,5,6*^

**Affiliations:**

^1^College of Advanced Interdisciplinary Studies, National University of Defense Technology, Changsha, Hunan 410073, China.

^2^Department of Optics and Optical Engineering, University of Science and Technology of China, Hefei, Anhui 230026, China.

^3^College of Intelligence Science and Technology, National University of Defense Technology, Changsha, Hunan 410073, China.

^4^State Key Laboratory of Low Dimensional Quantum Physics, Department of Physics, Tsinghua University, Beijing 100084, China.

^5^Department of Physics, Clarendon Laboratory, University of Oxford, Oxford, UK.

^6^School of Physical Science and Technology, ShanghaiTech University, Shanghai 201210, China.

^#^These authors contributed equally to this work.

^*^Email address: [c.zhang@nudt.edu.cn](mailto:c.zhang@nudt.edu.cn), huangk17@ustc.edu.cn, [yulin.chen@physics.ox.ac.uk](mailto:yulin.chen@physics.ox.ac.uk)

**This file contains the following sections and contents:**

**Section 1. One-dimensional real space scanning of a series of periodic grating with different widths.**

**Section 2. Luminescence spectrum excited by 177 nm laser.**

**Section 3. Methods for scanning imaging**

**Section 4. Discussion of energy and momentum resolution**

**Section 5. Focusing efficiency of the FZP**

**Section 6. Simulation of DOF of an ideal lens with the same N.A.**

**Figures S1~S7**

**Section 1. One-dimensional real space scanning of a series of periodic gratings with different widths**


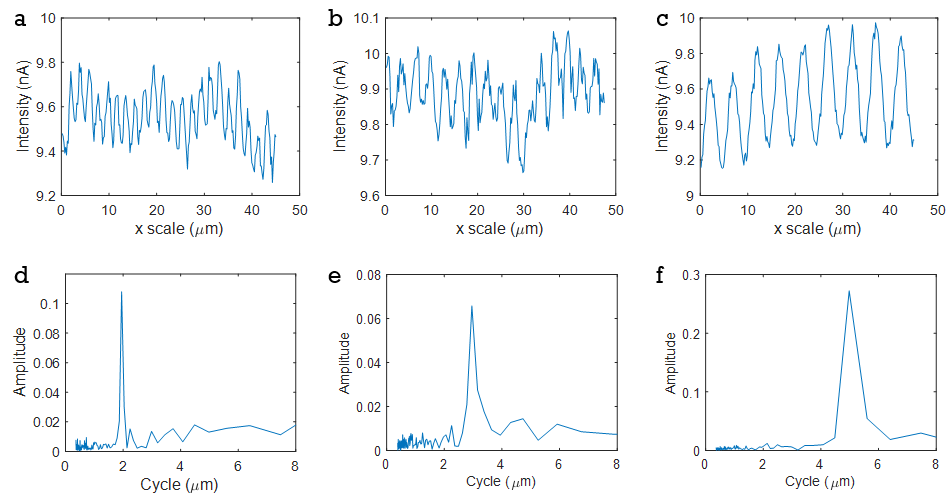


**Fig. S1.** Line-scanning images of periodic gratings in orthogonal orientations with 2 μm (**a**), 3 μm (**b**) and 5μm (**c**) period respectively. **d-f** give the fast Fourier transform of the line-scanning intensity corresponding to **a-c**


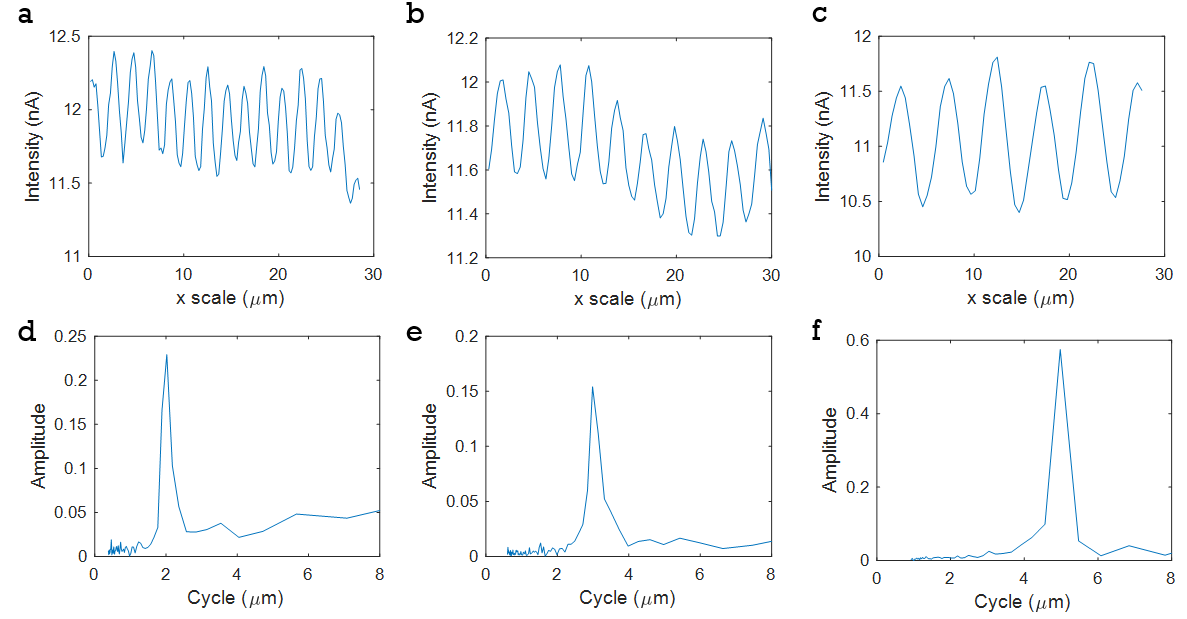


**Fig. S2.** Line-scanning images of periodic gratings in horizontal orientations with 2 μm (**a**), 3 μm (**b**) and 5μm (**c**) period respectively. **d-f** give the fast Fourier transform of the line-scanning intensity corresponding to **a-c**

**Section 2. Luminescence spectrum excited by 177 nm laser**


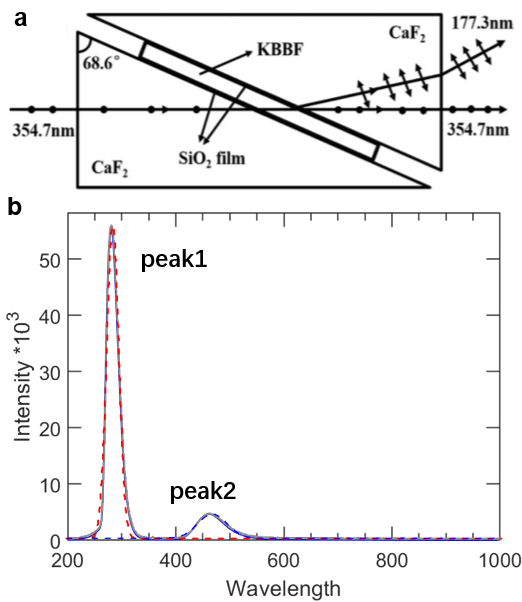


**Fig. S3. a.** Schematic of 177 nm laser emitted from KBBF crystal through a CaF_2_ prism; **b**. Luminescence spectrum of CaF_2_ excited by 177 nm laser, where the dashed lines are data fitting.

Considering the high photon energy of 177 nm laser (up to 7 eV), we believe that the optics devices made by CaF_2_, which are used to shape and focus the laser, will also emit fluorescence. When 177 nm laser is emitted from the KBBF crystal, it needs to be extracted by a CaF_2_ prism (as is shown in Fig S3(a)), which makes this fluorescence difficult to eliminate.

Fig. S3(b) shows luminescence spectrum of CaF_2_ excited by 177 nm laser. From the dashed line (data fitting with gaussian function), we can calculate features of the two peaks excited by 177 nm laser, which have been concluded in following table. Notably, we can see that the intensity ratio of **peak1** and **peak2** is about 12:1.

|  | **peak1** | **peak2** |
| --- | --- | --- |
| Position | 282 nm | 465 nm |
| FWHM | 26 nm | 64 nm |
| Intensity (a.u.) | 55448.8 | 1233.79 |

**Section 3. Methods for scanning imaging**

**1. Scanning transmission imaging of gratings and graphene/CaF_2_**


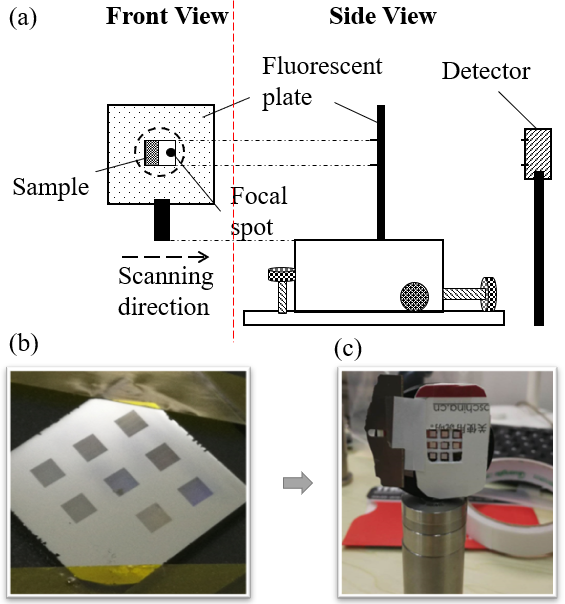


Fig. S4. Schematic of device for scanning transmission imaging. (a) The sketched setup for experimental measurement. (b-c) The graphene samples used in our work.

As shown in Figure S4(a), we placed the sample (grating or graphene with CaF2 substrate) at the center of a high-precision electric stage, which can move in the X/Y/Z directions with a minimum step of 0.1μm. The transmitted light through our sample is collected by a photodetector. The correlation between the stage and the photodetector must be made to record the detected signal for every movement of the stage, thus forming an image after the sample of interest is scanned. During the measurement, we firstly adjust the position of the stage along the propagation direction (i.e., z direction) of light so that we can observe the smallest and brightest hotspot. To facilitate the imaging process, we prepared some gratings with different periods from 50μm to 2μm. Then, the focal spot was illuminated onto the grating with the largest period for horizontal scanning, meanwhile the position of stage was finely tuned in Z direction. When the contrast of transmitted light through transparent parts and opaque parts is large enough, it indicates that the sample is located at the focal plane. Finally, the spot was moved to a grating with the smaller period gradually, so that the 1 micron-width grating was measured.

For the case of graphene/CaF_2_, we took the thick graphite as a knife edge to optimize the focal spot, and the similar steps as the scanning of the grating samples were used to achieve the scanning transmission image. The graphene flakes used in this work were prepared by a dry transfer method. The graphene was directly exfoliated from HOPG by a PDMS film, and then transferred to the CaF_2_ substrate. In order to find the target area easily in the measurement, we made a mark with a size of ～500μm near the target, and glued a fluorescent plate (made of very thin printing paper) around the marked area (Fig. S4(c)).

**2. Scanning imaging in ARPES**

Instead of photocurrent, scanning imaging in ARPES is achieved by visualization of the distribution of photoelectron with a certain energy. As shown in Figure S5, Photoelectrons are emitted from the sample by stimulation of focused VUV laser. Under the modulation of the electron lens and the deflection electric field, the photoelectrons reach the fluorescent screen through the hemispherical analyzer; and the camera behind the fluorescent screen can record these photoelectron signals. The photoemission experiment is carried out under ultra-high vacuum environment. Finally, through the two-dimensional scanning movement of the sample, we can achieve the image from ARPES.


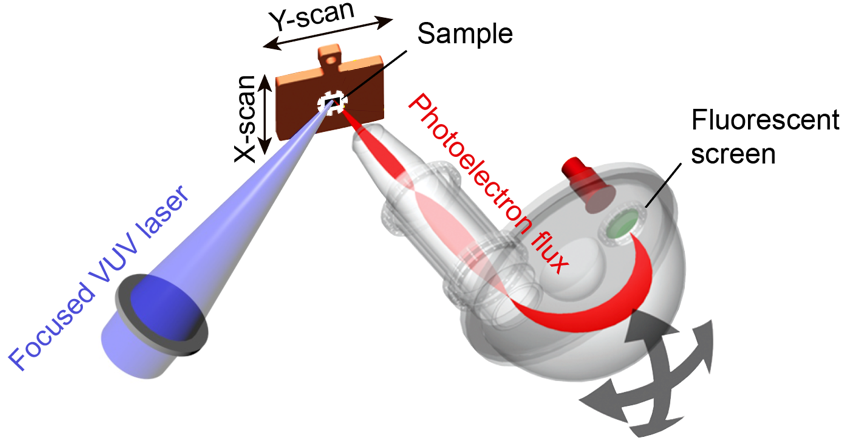


Fig. S5. Schematic of ARPES detecting process

**Section 4. Energy and momentum resolution**

In our system, the linewidth of 177nm VUV laser is about 0.26meV^1^, which can represent its energy resolution. Actually, the laser itself does not have an indicator of momentum resolution, however, in its application in ARPES system, the momentum resolution is given by^2^:

$\Delta k=\sqrt{\frac{2m_{e}E_{kin}}{\hbar}}sin\Delta\theta$ (S1)

According to the equation above, momentum resolution $\Delta k$ depends on angular resolution of the analyzer $\Delta\theta$ and the photoelectron kinetic energy $E_{kin}$. If $\Delta\theta$ is typically taken as 0.1° and $E_{kin}$ is 2.4eV^3^, we can achieve the momentum resolution is about 0.0028 Å^−1^.

In addition, the energy resolution in ARPES system has some connections with the spot size (space charge effect caused by high power density^4^), but mainly depends on the linewidth of the laser and performance of the ARPES analyzer. In the previously reported system with similar laser, the energy resolution is about 0.56meV^5^.

**Section 5. Focusing efficiency of the FZP**

To measure the focusing efficiency of the flat lens used in our paper, we make a whole-scale scan of the focal spot. As shown in Fig. S6, the intensity of the background light (dashed lines) is low. We obtained the experimental focusing efficiency of 40%, which is defined by the ratio of the power of the focal spot to the total power transmitted through the lens. It implies that the optical efficiency of this flat lens is 8% (=40%×20%), which approaches the theoretical efficiency limit of 10% for a binary-amplitude optical element.


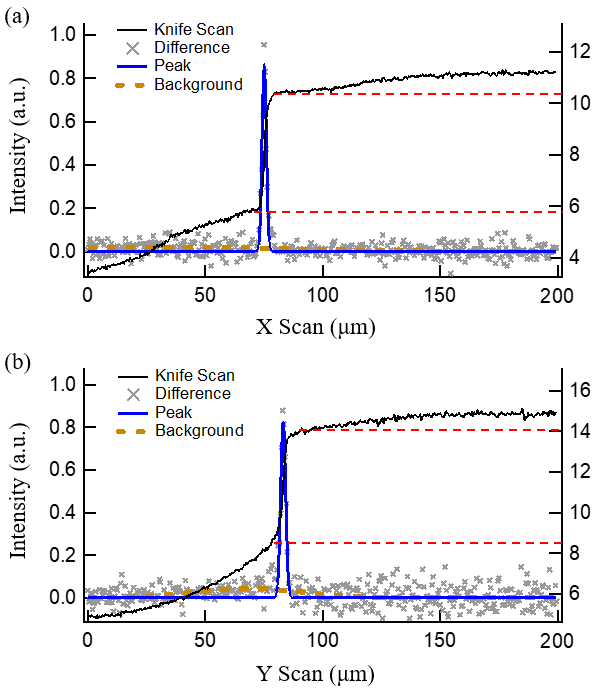


Fig. S6. Measurement of focusing efficiency along X/Y direction.

**Section 6. Simulation of DOF of an ideal lens with the same N.A.**

The maximum radius $r_{0}$ and focal length $f$ of our flat lens are 4895μm and 45000μm, respectively. It denotes a N.A. of about 0.108. Due to the small NA, the Fresnel diffraction under the paraxial approximation is used to simulate the focal field of an ideal spherical lens. The electric field$E_{out}\left( \xi,\eta\right)$ at the output plane is represented as:

$E_{out}\left( \xi,\eta\right)=\frac{e^{ikz}}{i\lambda z}\iint_{-\infty}^{+\infty} E_{in}\left( x,y \right)e^{i\frac{k}{2z}[\left( \xi-x \right)^{2}+{(\eta-y)}^{2}]}dxdy$ (S2)

Here, $E_{in}\left( x,y \right)=e^{-\frac{ik(x^{2}+y^{2})}{2f}}$ presents the phase profiles of the ideal spherical lens under the incident light with uniform illumination, $\lambda$=0.177μm is the wavelength, $f$=45000μm is the focal length,$k=\frac{2\pi}{\lambda}$ is the wave number. To calculate the DOF, only the on-axis $E_{out}\left( \xi,\eta\right)$ is employed, which means that $\xi$=0 and $\eta$=0. So we can get:

$E_{out}\left( 0,0 \right)=\frac{e^{ikz}}{i\lambda z}\iint e^{[i\frac{k}{2z}\left( x^{2}+y^{2} \right)-i\frac{k}{2f}\left( x^{2}+y^{2} \right)]}dxdy$ (S3)

This formula can be rewritten in polar coordinates shown as

$E_{out}\left( 0,0 \right)=\frac{e^{ikz}}{i\lambda z}\int_{0}^{r_{0}} e^{[i\frac{kr^{2}}{2z}-i\frac{kr^{2}}{2f}]}rdr\int_{0}^{2\pi} d\theta=\frac{{\pi r_{0}^{2}e}^{ikz}}{i\lambda z}\cdot\frac{sin[\frac{kr_{0}^{2}}{4}\left( \frac{1}{z}-\frac{1}{f} \right)]}{\frac{kr_{0}^{2}}{4}\left( \frac{1}{z}-\frac{1}{f} \right)}e^{i\frac{kr_{0}^{2}}{4}\left( \frac{1}{z}-\frac{1}{f} \right)}$ (S4)


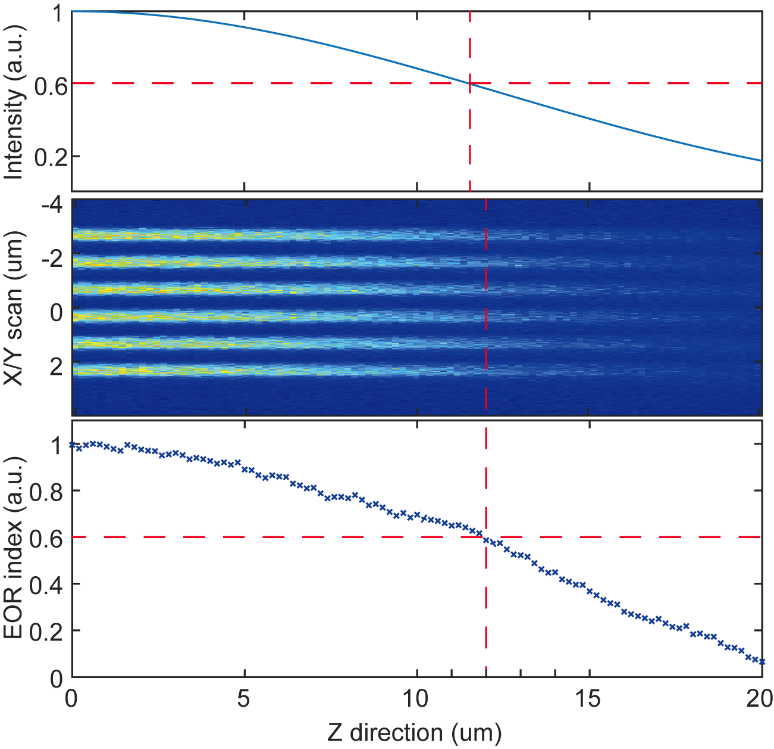


Fig. S7. Simulated DOF of ideal lens. (a) Depth of focus calculated by definition; (b) Simulated scan of grating by using ideal lens along Z direction and (c) its corresponding EOR index.

Figure S7(a) shows the simulated on-axis intensity along the z direction. It shows that the full width at its half maximum is about 23 microns. According to the method of measuring the DOF proposed in our work, the focal spot of the ideal spherical lens is simulated to scan a 2μm-period grating pattern, and 2% photocurrent noise was introduced in the simulation, see Fig. S7(b). According to the simulation results of the EOR Index, the effective optical resolution of 1μm-width stripe (DOF) of the ideal lens is about 24 microns, which is close to the standard definition result. Compared with the ideal spherical lens with the same NA, our flat lens offers a longer DOF, which facilitates the experimental implementation in the future applications of ARPES.

**Reference:**

1 Zhou X, He S, Liu G, Zhao L, Yu L, Zhang W. New developments in laser-based photoemission spectroscopy and its scientific applications: a key issues review. *Reports Prog Phys*; **81**, 062101 (2018).

2 Damascelli A, Hussain Z, Shen ZX. Angle-resolved photoemission studies of the cuprate superconductors. Rev. Mod. Phys. ; **75**, 473–541 (2003).

3 Chen YJ, Xu LX, Li JH, Li YW, Wang HY, Zhang CF *et al.* Topological Electronic Structure and Its Temperature Evolution in Antiferromagnetic Topological Insulator MnBi2Te4. *Phys Rev X*; **9**, 041040 (2019).

4 Hellmann S, Ott T, Kipp L, Rossnagel K. Vacuum space-charge effects in nano-ARPES. *Phys Rev B - Condens Matter Mater Phys*; **85**, 1–5 (2012).

5 Liu G, Wang G, Zhu Y, Zhang H, Zhang G, Wang X, Zhou Y, Zhang W, Liu H, Zhao L, Meng J, Dong X, Chen C, Xu Z, Zhou XJ. Development of a vacuum ultraviolet laser-based angle-resolved photoemission system with a superhigh energy resolution better than 1 meV. *Rev Sci Instrum*; **79**, 023105 (2008).
